# Somatic symptom severity and psychological distress among immigrants: findings from a population-based study

**DOI:** 10.1080/07853890.2025.2578726

**Published:** 2025-10-31

**Authors:** Eva M. Klein, Johannes Kruse, Bernd Löwe, Elmar Brähler, Claas Lahmann, Michael Witthöft, Lina Krakau

**Affiliations:** a Department of Psychosomatic Medicine and Psychotherapy, University Medical Center Freiburg, University of Freiburg, Freiburg, Germany; b Department of Psychosomatic Medicine and Psychotherapy, Justus Liebig University, Giessen, Germany; c Department of Psychosomatic Medicine and Psychotherapy, Centre for Internal Medicine, University Medical Centre Hamburg-Eppendorf, Hamburg, Germany; d Department of Psychosomatic Medicine and Psychotherapy, University Medical Center of the Johannes Gutenberg-University, Mainz, Germany; e Department of Clinical Psychology, Ruhr-University Bochum, Bochum, Germany

**Keywords:** Somatic symptom severity, SSS-8, immigrants, psychological distress, measurement invariance, population-based study

## Abstract

**Background:**

Somatic symptoms are frequently reported among immigrants, yet their occurrence and association with psychological distress remain understudied in population-based research using validated and measurement-invariant instruments. This study assessed the measurement invariance of the Somatic Symptom Scale-8 (SSS-8) and compared somatic symptom severity (SSS) between first- and second-generation immigrants and non-immigrants in a German population sample.

**Materials and methods:**

Somatic symptom severity and psychological distress were assessed using the SSS-8 and PHQ-4, respectively. Multi-group confirmatory factor analysis was conducted to test the measurement invariance of the SSS-8. Group differences were examined with ANOVA, and hierarchical regression analyses were performed to explore associations between immigration status and SSS, adjusting for distress and sociodemographic variables.

**Results:**

The sample included *N* = 6860 participants (46.8% women; *M* = 49.4 years, *SD* = 18.1), comprising *n* = 504 first-generation immigrants and *n* = 472 second-generation immigrants. The SSS-8 demonstrated measurement invariance across groups. Pain was the most frequently reported symptom. High or very high SSS was reported by 9.9% of first-generation immigrants, 12.7% of second-generation immigrants, and 7.5% of non-immigrants. In hierarchical regression models, second-generation immigrant status (*ß* = .15; 95% CI, 0.08–0.22; *p* < .001) was associated with greater SSS alongside higher age, female gender, lower income, and distress.

**Conclusions:**

In this study, the SSS-8 demonstrated measurement invariance across immigrant and non-immigrant populations, supporting its applicability for research in diverse sociocultural contexts. The findings suggest a higher vulnerability to SSS and distress among second-generation immigrants, emphasizing the need for targeted screening to improve early identification and healthcare access. However, clinical studies are needed to replicate these findings.

## Introduction

Somatic symptoms are highly prevalent in the general population [1,2], with ∼80% of individuals experiencing one or more symptoms within a four-week period [3]. Somatic symptoms can be defined as physical complaints that are subjectively perceived and reported by individuals, irrespective of whether a medical explanation is identified [c.f. 4]. Commonly reported symptoms include pain, sleep disturbance, and fatigue [5]. Somatic symptoms are a major reason for primary and specialist care consultations [6,7]. For approximately one third [3] to two thirds [7] of somatic symptoms presented in primary care, no clear biomedical explanation can be identified. Persistent somatic symptoms are a core feature of numerous medical conditions [8], are common in mental disorders, such as depression and anxiety [2,5,9], and represent a core diagnostic criterion for Bodily Distress Disorder (BDD) as defined in ICD-11, as well as for Somatic Symptom Disorder in DSM-5.

The prevalence of somatic symptoms varies across sociodemographic groups. While sociodemographic factors, such as older age, female gender, and lower socioeconomic status have been identified as risk factors [1,5,7,10], immigration status is often overlooked in epidemiological studies. Yet, there is a common assumption in psychiatric, psychosomatic, and general medical practice that non-Western immigrant groups tend to express emotional distress through somatic rather than psychological complaints [11]. Indeed, empirical studies have demonstrated that elevated somatic symptom severity is frequent in immigrant populations [12–14]. There is broad agreement, however, that immigrants may be at higher risk, not because of their immigration status per se, but due to migration-related factors, such as acculturative stress [15] and experiences of discrimination [16]. A systematic review reported that the prevalence of so-called somatization among immigrants ranged from 12.9 to 67% [17]. Research comparing somatic symptom severity in immigrants and the majority population, however, has shown inconsistent results. While some studies found a higher burden of somatic symptoms among immigrants [18–20], others reported similar levels compared with the majority population [21,22]. The inconsistent results raise a crucial question: Do these findings reflect real differences in the occurrence of somatic symptoms between immigrants and non-immigrants, or are they due to diagnostic bias or methodological problems with the questionnaire? In order to draw conclusions about differences in the prevalence of somatic symptom severity, the questionnaire used must measure the same construct, and the items must be interpreted consistently across groups to ensure measurement invariance—a prerequisite for valid estimates in comparative studies [23].

The Somatic Symptom Scale-8 (SSS-8), derived from the PHQ-15, is a widely used self-report measure of somatic symptom severity [24] and has been introduced as a short, time-efficient screening tool [25,26]. The SSS-8 assesses the presence and severity of the most frequent somatic symptoms, capturing somatic symptom burden as a subjective, self-reported experience, regardless of their cause [25]. A recent systematic review and meta-analysis investigating the measurement properties of the SSS-8 supported its use in assessing both general and domain-specific dimensions of somatic symptom severity, but also highlighted that cultural and linguistic differences can affect its measurement properties [27]. While measurement invariance of the PHQ-15 has been demonstrated in a small sample of immigrants [28], no study has addressed this issue for the SSS-8.

As persistent somatic symptoms are often overlooked, urgent action is recommended to implement standardized screening tools into routine assessments during medical consultations [8]. In addition, with increasing global migration, there is a strong need in clinical practice and research for valid, time-efficient tools that are applicable across sociocultural groups, as the assessment of somatic symptoms in transcultural contexts remains challenging due to presumed differences in illness perception and symptom presentation [17]. Given these challenges, studies should test the widespread clinical assumption that immigrants report higher levels of somatic symptom severity than the majority population, as this assumption may be influenced by stereotypes and requires cautious interpretation [29].

To address the research gap, the current study had two main aims. First, we tested the measurement invariance of the SSS-8 in a population-based sample to enable valid group comparisons. Second, we compared the occurrence of single somatic symptoms and the severity of somatic symptoms between first- and second-generation immigrants and non-immigrants, and we examined their association with psychological distress across the groups. Considering prior findings of differences in mental health outcomes and item loadings between first- and second-generation immigrants [30,31], we included generation status and controlled for established risk factors (gender, age, SES) to test for additional effects of immigration status.

## Materials and methods

### Sample and procedure

Data are based on three population-based surveys conducted in 2012, 2016, and 2021 by the independent demographic research institute USUMA Berlin. In accordance with the sampling guidelines for creating representative samples [32], a random-route procedure was used, in which regions, households, and the target person within the household were selected at random. Participants (*N* = 7549; 2012: *N* = 2510; 2016: *N* = 2524; 2021: *N* = 2515) completed self-report questionnaires at home in the presence of trained staff after giving consent. The analyzed sample (*N* = 6860) provided valid data on immigration status and was representative of the German general population in terms of age and gender.

### Sociodemographic data and questionnaires

Sociodemographic variables included age (≥14 years), gender (0 = male, 1 = female, 2 = diverse), high school diploma (0 = no, 1 = yes), and total household income. According to the OECD guidelines [33], equalized income was calculated (household income/√(people in household)). Participants were asked whether they and their parents were born in Germany. In line with previous studies [30], participants who reported that they had been born outside Germany, with at least one parent who was also born outside Germany, were defined as first-generation immigrants. Participants who reported being born in Germany with at least one immigrant parent were considered as second-generation immigrants.

### Somatic symptom severity

Somatic symptom severity was assessed with the Somatic Symptom Scale-8 (SSS-8) [25]. The eight items cover four subscales: pain, fatigue, gastrointestinal, and cardiopulmonary symptoms. Symptoms over the last seven days were rated on a 5-point Likert scale (0 = not at all to 4 = very much), with total scores ranging from 0 to 32. The SSS-8 showed good psychometric properties [25,26] with Cronbach’s *α* = .84 in the current study. A cut-off score ≥12 indicates high and very high somatic symptom severity [25].

### Psychological distress (PHQ-4)

The PHQ-4 [34] assesses symptoms of depression and anxiety with two items each, rated on a Likert scale from 0 (not at all) to 3 (nearly every day), resulting in a total score ranging from 0 to 12. In this study, the scale demonstrated good internal consistency (Cronbach’s *α* = .86), and measurement invariance across immigrant samples has been confirmed in a recent study [31].

### Ethical approval statement

The survey and its procedures were approved by the institutional ethics review board of the University of Leipzig (reference numbers: 2012: 092-12-05032012; 2016: 452-15-21122015; 2021: 298/21-ek). The study was conducted in accordance with the Declaration of Helsinki and relevant institutional ethics guidelines. The surveys adhered to the principles of ICH-GCP and the ICC/ESOMAR International Code of Marketing and Social Research Practice.

Participants received detailed information about the study procedure, data handling, anonymization, and data protection before providing verbal informed consent. In the case of underage participants, legal guardians were informed first and provided verbal consent for their child’s participation. Subsequently, the minors themselves also gave verbal consent. Participation was voluntary, and all participants were informed about their data protection rights in advance. No audio or video recording systems were used to document verbal consent, in accordance with ethical approval. According to the German Federal Data Protection Act (§4a BDSG), verbal consent is sufficient for fully anonymized social and marketing research that is not subject to the European Union General Data Protection Regulation (GDPR). Written consent was not required due to the anonymous nature of the data collected. The verbal consent procedure was explicitly approved by the Ethics Committee of the University of Leipzig.

### Statistical analyses

To test the measurement invariance of the SSS-8, we conducted a confirmatory factor analysis (CFA) on the total sample and on the three subsamples (first-generation immigrants, second-generation immigrants, and non-immigrants). We adapted the factor structure reported by Gierk et al. 2014 [25] with a higher-order general factor and four subdimensions of pain (items 2, 3, 4), fatigue (items 7, 8), cardiopulmonary (items 5, 6), and gastrointestinal symptoms (item 1). We applied robust maximum likelihood estimation (MLR in R/lavaan) used common fit statistics to evaluate model fit [35]: (1) absolute fit indices (an exact-fit test of model chi-square and the standardized root mean square residual [SRMR]) and (2) incremental fit indices (root mean square error of approximation [RMSEA] and the comparative fit index [CFI]). Values smaller than .08 for the SRMR and RMSEA, and >.95 for CFI indicate an acceptable fit [36]. Next, we used multi-group CFA [37] to evaluate measurement invariance across immigration samples. We tested for configural invariance (without constraints), metric invariance (factor loadings constrained to be equal across groups), scalar invariance (factor loadings and intercepts constrained to be equal across groups), and strict invariance (factor loadings, intercepts, and residuals constrained to be equal across groups). Models were accepted as invariant if the differences in CFI, RMSEA, and SRMR were ≤0.01 [38]. Differences in somatic symptom severity according to immigration status were compared using three-way analysis of variance with the SSS-8 total score as the dependent variable, gender, and immigration status included as factors. Main effects and the interactions between all factors were examined. Significant effects were analyzed using Bonferroni-corrected Tukey post hoc analysis. Finally, to investigate the association of somatic symptom severity with immigration status beyond psychological distress and established sociodemographic risk factors, we conducted multiple hierarchical regression analyses. ANOVA and regression analyses were controlled for the different assessment points. Missing data on variables relevant to our analysis ranged between 0 and 3.2% and were handled using listwise deletion. We conducted all statistical analyses in R, using the packages lavaan and semTools [39] and psych [40].

## Results

### Factor structure and measurement invariance

Confirmatory factor analysis (CFA) showed an acceptable to very good model fit for the total sample [*X*^2^(*df*) = 437.84 (17), *p* = 0.001; CFI = 0.956, RMSEA = 0.084, SRMR = 0.047], non-immigrants [*X*^2^(*df*) = 357.91 (17), *p* = 0.001, CFI = 0.957, RMSEA = 0.082; SRMR = 0.047], and second-generation immigrants [*X*^2^(*df*) = 44.19 (17), *p* = 0.001, CFI = 0.965; RMSEA = 0.079; SRMR = 0.042]. In first-generation immigrants, RMSEA exceeded the cut-off (RMSEA = 1.00), while CFI and SRMR did not [First Gen: *X*^2^(*df*) = 64.54 (17), *p* = 0.001, CFI = 0.945, SRMR = 0.053]. Therefore, the model was judged as acceptable. Based on multigroup CFA, we found strict factorial invariance to hold across groups (Table 1). Hence, the SSS-8 can be considered measurement invariant across first-generation, second-generation immigrants, and non-immigrants.

**Table 1.** Test for invariance across immigration status.

	*X*^2^ (*df*)	*p*	Δ*Χ*^2^ (Δ*df*)	*p*	CFI	ΔCFI	RMSEA	ΔRMSEA	SRMR	ΔSRMR
Configural model	483.32 (51)	<0.001			0.956		0.083		0.043	
Metric model	488.74 (65)	<0.001	50.42 (14)	0.002	0.956	0.000	0.074	0.009	0.045	0.002
Scalar model	526.70 (73)	<0.001	37.96 (8)	0.023	0.955	0.001	0.070	0.004	0.045	0.000
Strict model	531.65 (89)	<0.001	16 (4.95)	<0.001	0.953	0.003	0.066	0.005	0.047	0.002

### Comparison between immigrant groups and non-immigrants

**Figure 1.**
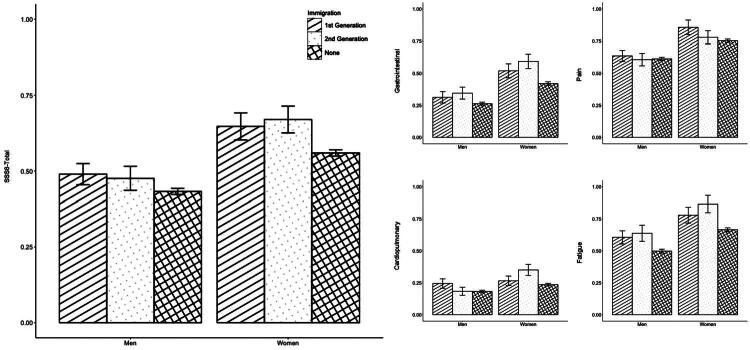
Mean and Standard Error of the SSS-8 Total Score and Subscales stratified by Immigration Status and Gender

Table 2 shows the sociodemographic characteristics of the sample stratified by immigration status. Regardless of immigration status, pain was the leading symptom, followed by fatigue, gastrointestinal, and cardiopulmonary symptoms. Non-immigrants reported fewer somatic symptoms on both the total and subscales of the SSS-8 compared to first- and second-generation immigrants. Accordingly, a higher proportion of first- and second-generation immigrants reported high or very high levels of somatic symptoms. Descriptively, second-generation immigrants showed the highest somatic symptom severity, particularly in the subscales of fatigue, cardiopulmonary, and gastrointestinal symptoms. Pain, however, was more frequently reported by first-generation immigrants. While the difference in average symptom scores was small (*η*^2^ = 0.002), it became more pronounced when comparing groups with high and very high symptom severity (*V* = 0.052), suggesting a difference in the number of people reporting clinically relevant levels of somatic symptom distress according to immigration status. Immigrants reported also higher levels of psychological distress. Regarding gender, women scored significantly higher on the total scale (*M* = 0.57; *SD* = 0.60) compared to men (*M* = 0.44; *SD* = 0.54). Figure 1 displays the mean and standard error of the SSS-8 total score and its subscales, stratified by immigration status and gender.

**Table 2.** Sample characteristics for the total sample and stratified by immigration status.

		Total sample	1st Generation immigrants	2nd Generation immigrants	Non- immigrant	*p*	*η*^2^/*V*
*N*		6860	504	472	5884		
Gender No. (%)	Non-binary	1 (0.0)	0 (0.0)	0 (0.0)	1 (0.0)	0.205	
	Female	3532 (46.8)	263 (52.2)	230 (48.7)	2754 (46.8)		
	Male	4016 (53.2)	241 (47.8)	242 (51.3)	3129 (53.2)		
Age *M* (*SD*)		49.43 (18.08)	46.83 (16.74)	40.46 (17.97)	50.10 (17.98)	<0.001	0.02
High school education No. (%)	Yes	1544 (20.5)	123 (24.4)	103 (21.8)	1177 (20.0)	0.049	0.029
	No	6002 (79.5)	381 (75.6)	368 (78.0)	4705 (80.0)		
Equivalized income *M* (*SD*)		1783.23 (921.56)	1592.69 (815.41)	1755.72 (968.84)	1801.73 (924.49)	<0.001	0.004
Distress (PHQ-4)		1.39 (2.02)	1.57 (2.09)	1.61 (2.21)	1.35 (1.99)	0.003	0.002
Somatic symptoms (SSS-8) *M* (*SD*)		0.51 (0.57)	0.57 (0.63)	0.58 (0.65)	0.50 (0.56)	0.002	0.002
SSS-8 gastro-intestinal *M* (*SD*)		0.36 (0.71)	0.41 (0.78)	0.47 (0.80)	0.35 (0.70)	<0.001	0.002
SSS-8 pain *M* (*SD*)		0.69 (0.73)	0.74 (0.79)	0.70 (0.77)	0.69 (0.72)	0.322	
SSS-8 cardiopulmonary *M* (*SD*)		0.22 (0.53)	0.26 (0.58)	0.27 (0.59)	0.21 (0.52)	0.029	0.001
SSS-8 fatigue *M* (*SD*)		0.61 (0.85)	0.69 (0.89)	0.75 (1.00)	0.59 (0.83)	<0.001	0.003
SSS-8 total cut-off No. (%)	Yes		50 (9.9)	60 (12.7)	440 (7.5)	<0.001	0.052
	No		443 (87.9)	407 (86.2)	5327 (90.5)		

*V*: Cramér’s *V*.

Based on analysis of variance with the SSS-8 score as dependent variable, we found a significant effect of immigration status [*F*(2, 6708) = 6.165, *p* = 0.002, *η*^2^ = 0.002] and gender [*F*(1, 6708) = 93.96, *p* < .001, *η*^2^ = 0.01]. First-generation (−0.07, *p* = 0.020) and second-generation immigrants (−0.08, *p* < .035) had higher SSS-8 scores compared to non-immigrants. Yet, the effect size of immigration status was negligible. Women reported a significantly higher symptom severity compared to men (0.13, *p* < .001), regardless of immigration status. No statistically significant interactions were found between gender and immigration status [*F*(2, 6706) = 0.49, *p* = 0.608].

### Regression analysis

The results of the regression analysis with the SSS-8 total score as dependent variable and sociodemographic factors and psychological distress as independent variables are shown in Table 3. Higher age, female gender, and lower household income were associated with higher somatic symptom severity. Second-generation immigrants reported higher somatic symptom severity compared to non-immigrants. Without controlling for psychological distress, this also applied to first-generation immigrants. Including immigration status resulted in a small but significant increase in explained variance (model 3) compared to a model with other sociodemographic factors (model 2). Psychological distress was significantly associated with somatic symptom severity and provided the greatest increase in explained variance. No significant interactions were found between immigration status and psychological distress (model 4).

**Table 3.** Results from hierarchical regression analysis with somatic symptom severity as outcome.

	Model 1				Model 2				Model 3				Model 4			
				95%CI				95%CI				95%CI				95%CI
	*ß*	*SE*	*p*	LL;UL	*ß*	*SE*	*p*	LL;UL	*ß*	*SE*	*p*	LL;UL	*ß*	*SE*	*p*	LL;UL
Intercept	−0.31	0.02	**<0.001**	−0.36; 0.27	−0.32	0.03	**<0.001**	−0.38; −0.25	−0.35	0.03	**<0.001**	−0.41; −0.28	−0.23	0.03	**<0.001**	−0.28; −0.18
Gender	0.21	0.02	**<0.001**	0.16; 0.25	0.19	0.02	**<0.001**	0.14; 0.23	0.19	0.02	**<0.001**	0.14; 0.23	0.11	0.02	**<0.001**	0.07; 0.14
Age	0.30	0.01	**<0.001**	0.27; 0.32	0.30	0.01	**<0.001**	0.27; 0.32	0.31	0.01	**<0.001**	0.28; 0.33	0.21	0.01	**<0.001**	0.19; 0.23
Equalized income					−0.13	0.01	**<0.001**	−0.16; −0.11	−0.13	0.01	**<0.001**	−0.15; −0.11	−0.04	0.01	**<0.001**	−0.06; −0.02
High school education					−0.01	0.03	0.702	−0.07; 0.05	−0.01	0.03	0.765	−0.07; 0.05	0.03	0.01	0.231	−0.02; 0.07
1st Generation									0.13	0.04	**0.002**	0.05; 0.22	0.06	0.03	0.062	−0.00; 0.13
2nd Generation									0.27	0.05	**<0.001**	0.18; 0.36	0.15	0.05	**<0.001**	0.08; 0.22
Distress													0.60	0.01	**<0.001**	0.58; 0.62
1st Gen*Distress													0.02	0.03	0.520	−0.04; 0.09
2nd Gen*Distress													0.06	0.03	0.063	−0.00; 0.12
	Adj. *R*^2^ = 0.121				Adj. *R*^2^ = 0.137				Adj. *R*^2^ = 0.142				Adj. *R*^2^ = 0.487			
					Model 2 *vs.* Model 1				Model 3 *vs.* Model 2				Model 4 *vs.* Model 3			
					Test *F* = 63.69 (2, 6489), *p* < .001				Test *F* = 35.60 (2, 6485), *p* < .001				Test *F* = 1455.6 (3, 6482), *p* < .001			

*Note.* All Analyses were controlled for the different assessment points (0 = 2012; 1 = 2016; 2 = 2021); 0 = non-immigrant, 1 = 1st generation immigrant; 2nd generation 2: 0 = non-immigrant, 1 = 2nd generation immigrants.

## Discussion

In this study, we assessed somatic symptom severity and its association with psychological distress across immigrant groups (first- and second-generation immigrants) and non-immigrants in the general population, ensuring valid comparisons by first testing the measurement invariance of SSS-8. Immigrants reported greater somatic symptom severity than the majority population, with a small effect size, after controlling for other sociodemographic risk factors. As the SSS-8 demonstrated measurement invariance across groups, these differences likely reflect true variations rather than measurement bias. Somatic symptom severity was associated with higher psychological distress across all groups. The findings suggest that immigrants report both somatic and psychological symptoms, rather than following a pattern of ‘somatization’ in which somatic symptoms are typically reported instead of psychological symptoms. The results thus contradict the widespread assumption that immigrants tend to express distress differently from the majority population [41].

The primary aim of our study was to assess the measurement invariance of the SSS-8 across immigrant groups as a prerequisite for valid comparisons. Our analyses confirmed measurement invariance for the SSS-8 in a population-based sample, aligning with previous findings on the PHQ-15 in a smaller immigrant sample [28], in populations of Syrian refugees in Germany [42], and in adolescents with an immigration history [43].

Regarding the occurrence of somatic symptoms, pain was the most commonly reported symptom across all groups, followed by fatigue, gastrointestinal, and cardiopulmonary symptoms. This is consistent with previous findings in the general population, where different forms of pain are frequently reported as the leading symptom [5,44]. Regardless of immigration status, somatic symptom severity was strongly associated with psychological distress, supporting the well-established Somatic-Anxiety-Depressive Triade [9,24,45].

Compared to the majority population, first- and second-generation immigrants reported both higher somatic symptom severity and psychological distress. Although the effect sizes were small, immigration status systematically contributed to the variance in somatic symptoms, highlighting it as a sociodemographic risk factor. This finding is consistent with previous research indicating greater vulnerability to psychological distress among immigrants [30], which arises not from immigration status per se but from the interplay of systemic disadvantages, migration-related stressors, and contextual conditions, thereby underscoring the multifactorial nature of somatic symptom severity [46]. In the post-migration context, immigrants are often exposed to socioeconomic disadvantages, such as unemployment, low income, and lower educational attainment, which are well-established risk factors for poor health outcomes [47], including somatic symptom burden [1,5]. A recent mixed-methods study, for example, found that post-migration stressors, such as economic insecurity are linked to increased psychological distress, which in turn heightens the risk of somatic symptoms [48]. Studies also suggest an association between acculturation and the prevalence of somatic symptoms [49], although the direction of this relationship remains inconsistent [17]. Healthcare-related factors also play a role, as studies reveal access barriers to mental health services [50,51], mainly due to language barriers, discrimination, and limited information about available services [52]. This is particularly relevant in the context of the severity of somatic symptoms, where timely access to mental healthcare is important to address subjective distress and reduce the risk of chronicity and severe outcomes [53]. Immigrants may also experience more stressful life events, a known predictor of somatic symptoms in the general population [54]. For instance, racial discrimination is a pervasive stressor [16] that is associated with somatic, anxiety, and depressive symptoms in a striking dose-response manner [55]. Conversely, supportive social and societal environments that foster inclusion can buffer the mental health risks faced by immigrants [56], highlighting the importance of the social climate toward immigrants.

In our study, second-generation immigrants showed clinically relevant levels of somatic distress, particularly in fatigue and cardiopulmonary symptoms. Even after controlling for other risk factors, they exhibited the highest symptom levels. In contrast to previous studies that reported higher somatic symptom severity among first-generation Turkish immigrants and particularly among Turkish women [19], our findings suggest higher somatic symptom severity and greater distress in the second-generation, without gender-specific interactions. Prior research has also highlighted generational differences in mental health outcomes [30], yet second-generation immigrants are often neglected in research, including reviews on somatization that focus exclusively on first-generation immigrants [17]. Higher somatic symptom severity and distress in second-generation immigrants may be attributable to the distinctive challenges associated with growing up between two cultures, contributing to family and intergenerational conflict and identity disturbance, the latter of which has been linked to somatic and emotional distress [57].

Immigrants reported both greater somatic symptom severity and distress, challenging the notion that they express psychological distress primarily through somatic symptoms [11,41]. Similarly, clinical studies have found increased somatic symptoms in immigrants without a corresponding decrease in depressive symptoms [58,59]. We found no significant differences in the experience of pain, reinforcing the need for caution in attributing differences in pain solely to cultural factors [29,41]. While pain has often been discussed in terms of cultural idiosyncrasies [29,60], country-level variables, such as the Gender Inequality Index better explain differences in pain prevalence in cross-cultural research.

### Limitations

A strength of this study is its large sample size, but a key limitation is the under-representation of immigrants compared to their proportion in the general population, a common issue in population-based studies of immigrant mental health [61]. The use of questionnaires only available in German may have biased the sample toward immigrants with better language skills. This limits the generalizability of the results, as immigrants with limited language proficiency reported higher symptom levels on the PHQ-15 than those with better language skills [19,58]. Migration-specific data, such as country of origin and reasons for migration were not assessed. As a result, the sample of immigrants is likely to be heterogeneous and grouping them together may mask important sociocultural differences. Despite this limitation, the large sample size helps balance out group-specific characteristics, enhancing ecological validity. Future studies should test the measurement invariance of the SSS-8 among immigrants from specific national or linguistic backgrounds and identify psychological and behavioral risk factors for somatic symptoms, particularly to better understand the higher somatic symptom severity among second-generation immigrants. Furthermore, future research should examine acculturative stress as a moderator to better clarify the link between somatic symptom severity and immigration status.

## Conclusion

Compared with the majority population, immigrants reported greater severity of somatic symptoms and distress, although the effect sizes were small. The German version of the SSS-8 demonstrates measurement invariance across immigrant populations and is suitable for comparative mental health research. Implications for public health include the suitability of the SSS-8 as a valid screening tool for early identification of vulnerable groups, particularly as somatic symptoms predict health outcomes beyond anxiety, depression, and other medical conditions [6]. Targeted screening for somatic symptoms could help identify health risks early, potentially reducing the marginalization of immigrants in pain management [62] and improving access to appropriate care. Further clinical samples are needed to validate the new ICD-11 criteria for Somatic Symptom Disorder (SSD) in immigrant populations, a largely neglected area to date [63].

## Data Availability

The data of this study are available from the corresponding author upon reasonable request.

## References

[1] Beutel ME, Klein EM, Henning M, et al. Somatic symptoms in the German general population from 1975 to 2013. Sci Rep. 2020;10(1):1595. doi: 10.1038/s41598-020-58602-6. 32005895 PMC6994459

[2] Creed FH, Davies I, Jackson J, et al. The epidemiology of multiple somatic symptoms. J Psychosom Res. 2012;72(4):311–317. doi: 10.1016/j.jpsychores.2012.01.009. 22405227

[3] Kroenke K. Patients presenting with somatic complaints: epidemiology, psychiatric co-morbidity and management. Int J Methods Psychiatr Res. 2003;12(1):34–43. doi: 10.1002/mpr.140. 12830308 PMC6878426

[4] Lehmann M, Pohontsch NJ, Zimmermann T, et al. Diagnostic and treatment barriers to persistent somatic symptoms in primary care–representative survey with physicians. BMC Fam Pract. 2021;22(1):60. doi: 10.1186/s12875-021-01397-w. 33794776 PMC8017612

[5] Hinz A, Ernst J, Glaesmer H, et al. Frequency of somatic symptoms in the general population: normative values for the Patient Health Questionnaire-15 (PHQ-15). J Psychosom Res. 2017;96:27–31. doi: 10.1016/j.jpsychores.2016.12.017. 28545789

[6] Tomenson B, Essau C, Jacobi F, et al. Total somatic symptom score as a predictor of health outcome in somatic symptom disorders. Br J Psychiatry. 2013;203(5):373–380. doi: 10.1192/bjp.bp.112.114405. 24072756

[7] Steinbrecher N, Koerber S, Frieser D, et al. The prevalence of medically unexplained symptoms in primary care. Psychosomatics. 2011;52(3):263–271. doi: 10.1016/j.psym.2011.01.007. 21565598

[8] Toussaint A, Weigel A, Löwe B, et al. The overlooked burden of persistent physical symptoms: a call for action in European healthcare. Lancet Reg Health Eur. 2025;48:101140. 39660101 10.1016/j.lanepe.2024.101140 PMC11629243

[9] Löwe B, Spitzer RL, Williams JB, et al. Depression, anxiety and somatization in primary care: syndrome overlap and functional impairment. Gen Hosp Psychiatry. 2008;30(3):191–199. doi: 10.1016/j.genhosppsych.2008.01.001. 18433651

[10] Barsky AJ, Peekna HM, Borus JF. Somatic symptom reporting in women and men. J Gen Intern Med. 2001;16(4):266–275. doi: 10.1046/j.1525-1497.2001.016004266.x. 11318929 PMC1495200

[11] Erim Y, Morawa E. Somatisierung und somatoforme Störungen. In: Praxis der Interkulturellen Psychiatrie und Psychotherapie. Munich, Germany: Elsevier; 2018. p. 417–425.

[12] Aragona M, Rovetta E, Pucci D, et al. Somatization in a primary care service for immigrants. Ethn Health. 2012;17(5):477–491. doi: 10.1080/13557858.2012.661406. 22352805

[13] Aragona M, Tarsitani L, Colosimo F, et al. Somatization in primary care: a comparative survey of immigrants from various ethnic groups in Rome, Italy. Int J Psychiatry Med. 2005;35(3):241–248. doi: 10.2190/2G8N-MNNE-PGGP-PJJQ. 16480239

[14] Rask S, Suvisaari J, Koskinen S, et al. The ethnic gap in mental health: a population-based study of Russian, Somali and Kurdish origin migrants in Finland. Scand J Public Health. 2016;44(3):281–290. doi: 10.1177/1403494815619256. 26647096

[15] Lerias D, Ziaian T, Miller E, et al. The role of acculturative stress on the mental health of immigrant youth: a scoping literature review. Community Ment Health J. 2025;61(3):462–491. doi: 10.1007/s10597-024-01351-x. 39240483 PMC11868275

[16] Schunck R, Reiss K, Razum O. Pathways between perceived discrimination and health among immigrants: evidence from a large national panel survey in Germany. Ethn Health. 2015;20(5):493–510. doi: 10.1080/13557858.2014.932756. 24992379

[17] Lanzara R, Scipioni M, Conti C. A clinical-psychological perspective on somatization among immigrants: a systematic review. Front Psychol. 2018;9:2792. doi: 10.3389/fpsyg.2018.02792. 30705662 PMC6344401

[18] Bragazzi NL, Puente GD, Natta WM. Somatic perception, cultural differences and immigration: results from administration of the Modified Somatic Perception Questionnaire (MSPQ) to a sample of immigrants. Psychol Res Behav Manag. 2014;7:161–166. doi: 10.2147/PRBM.S55393. 24966706 PMC4062560

[19] Morawa E, Dragano N, Jöckel K-H, et al. Somatization among persons with Turkish origin: results of the pretest of the German National Cohort Study. J Psychosom Res. 2017;96:1–9. doi: 10.1016/j.jpsychores.2017.02.014. 28545785

[20] Salinero-Fort MA, Jiménez-García R, de Burgos-Lunar C, et al. Common mental disorders in primary health care: differences between Latin American-born and Spanish-born residents in Madrid, Spain. Soc Psychiatry Psychiatr Epidemiol. 2015;50(3):429–443. doi: 10.1007/s00127-014-0962-5. 25273551

[21] Bermejo I, Nicolaus L, Kriston L, et al. Vergleichende Analyse psychosomatischer Beschwerden bei Personen mit spanischem, italienischem, türkischem und russischem Migrationshintergrund. Psychiatr Prax. 2012;39(4):157–163. [Mismatch ] 22334132 10.1055/s-0031-1298903

[22] Miranda J, Siddique J, Belin TR, et al. Depression prevalence in disadvantaged young black women: African and Caribbean immigrants compared to US-born African Americans. Soc Psychiatry Psychiatr Epidemiol. 2005;40(4):253–258. doi: 10.1007/s00127-005-0879-0. 15834775

[23] Dimitrov DM. Testing for factorial invariance in the context of construct validation. Meas Eval Counsel Dev. 2010;43(2):121–149. doi: 10.1177/0748175610373459.

[24] Kroenke K, Spitzer RL, Williams JB. The PHQ-15: validity of a new measure for evaluating the severity of somatic symptoms. Psychosom Med. 2002;64(2):258–266. doi: 10.1097/00006842-200203000-00008. 11914441

[25] Gierk B, Kohlmann S, Kroenke K, et al. The Somatic Symptom Scale-8 (SSS-8): a brief measure of somatic symptom burden. JAMA Intern Med. 2014;174(3):399–407. doi: 10.1001/jamainternmed.2013.12179. 24276929

[26] Gierk B, Kohlmann S, Toussaint A, et al. Assessing somatic symptom burden: a psychometric comparison of the Patient Health Questionnaire-15 (PHQ-15) and the Somatic Symptom Scale-8 (SSS-8). J Psychosom Res. 2015;78(4):352–355. doi: 10.1016/j.jpsychores.2014.11.006. 25498316

[27] Hybelius J, Kosic A, Salomonsson S, et al. Measurement properties of the Patient Health Questionnaire-15 and Somatic Symptom Scale-8: a systematic review and meta-analysis. JAMA Netw Open. 2024;7(11):e2446603. doi: 10.1001/jamanetworkopen.2024.46603. 39565620 PMC11579800

[28] Mewes R, Christ O, Rief W, et al. Sind Vergleiche im Depressions-und Somatisierungsausmaß zwischen Migranten und Deutschen möglich. Diagnostica. 2010;56(4):230–239. doi: 10.1026/0012-1924/a000026.

[29] Benabdeljlil M. Somatization and functional disorders in migrants and refugees. In: Neurology in migrants and refugees. Berlin, Germany: Springer; 2022. p. 309–322.

[30] Beutel ME, Jünger C, Klein EM, et al. Depression, anxiety and suicidal ideation among 1 st and 2 nd generation migrants-results from the Gutenberg health study. BMC Psychiatry. 2016;16(1):288. doi: 10.1186/s12888-016-0995-2. 27516075 PMC4982128

[31] Tibubos AN, Beutel ME, Schulz A, et al. Is assessment of depression equivalent for migrants of different cultural backgrounds? Results from the German population-based Gutenberg Health Study (GHS). Depress Anxiety. 2018;35(12):1178–1189. doi: 10.1002/da.22831. 30156742

[32] Koch A. ADM-Design und Einwohnermelderegister-Stichprobe. In: Gabler S, Hoffmeyer-Zlotnik J, editors. Stichproben in der Umfragepraxis. Opladen: Springer; 1997. p. 99–116.

[33] OECD. An overview of growing income inequalities in OECD countries: Main findings. Available at: http://www.oecd.org/els/soc/49499779.pdf

[34] Löwe B, Wahl I, Rose M, et al. A 4-item measure of depression and anxiety: validation and standardization of the Patient Health Questionnaire-4 (PHQ-4) in the general population. J Affect Disord. 2010;122(1–2):86–95. doi: 10.1016/j.jad.2009.06.019. 19616305

[35] West SG, Wu W, McNeish D, et al. Model fit in structural equation modeling. In: Hoyle RH, editor. Handbook of structural equation modeling; 2023. p. 184–205.

[36] Schermelleh-Engel K, Moosbrugger H, Müller H. Evaluating the fit of structural equation models: tests of significance and descriptive goodness-of-fit measures. Methods Psychol Res Online. 2003;8:23–74.

[37] Meredith W. Measurement invariance, factor analysis and factorial invariance. Psychometrika. 1993;58(4):525–543. doi: 10.1007/BF02294825.

[38] Rensvold RB, Cheung GW. Testing for metric invariance using structural equation models, solving the standardization problem. Res Manag. 2001;1:25–50.

[39] Jorgensen TD, Pornprasertmanit S, Schoemann AM, et al. semTools: useful tools for structural equation modeling (0.5-3) [Computer software]; 2020. Available from: https://CRAN.R-project.org/package=semTools

[40] Revelle W. psych: procedures for psychological, psychometric, and personality research. R package version 2; 2020.

[41] Gureje O. What can we learn from a cross-national study of somatic distress? J Psychosom Res. 2004;56(4):409–412. doi: 10.1016/S0022-3999(03)00623-8. 15094024

[42] Schlechter P, Hellmann JH, Morina N. Assessing somatic symptoms with the Patient Health Questionnaire (PHQ-15) in Syrian refugees. Assessment. 2023;30(4):1211–1225. doi: 10.1177/10731911221086986. 35450445 PMC10152221

[43] Kliem S, Krieg Y, Beller J, et al. Psychometric properties of the Somatic Symptom Scale 8 (SSS-8) in a representative sample of German adolescents. J Psychosom Res. 2021;149:110593. doi: 10.1016/j.jpsychores.2021.110593. 34371255

[44] Hiller W, Rief W, Brähler E. Somatization in the population: from mild bodily misperceptions to disabling symptoms. Soc Psychiatry Psychiatr Epidemiol. 2006;41(9):704–712. doi: 10.1007/s00127-006-0082-y. 16794766

[45] Smakowski AL, Hüsing P, Völcker S, et al. Psychological risk factors of somatic symptom disorder: a systematic review and meta-analysis of cross-sectional and longitudinal studies. J Psychosom Res. 2024;181:111608. doi: 10.1016/j.jpsychores.2024.111608. 38365462

[46] Löwe B, Toussaint A, Rosmalen JGM, et al. Persistent physical symptoms: definition, genesis, and management. Lancet. 2024;403(10444):2649–2662. doi: 10.1016/S0140-6736(24)00623-8. 38879263

[47] Hynie M. The social determinants of refugee mental health in the post-migration context: a critical review. Can J Psychiatry. 2018;63(5):297–303. doi: 10.1177/0706743717746666. 29202665 PMC5912301

[48] Blanton M. Post-migration stressors and mental and physical health among refugees and migrants in need of protection: a mixed-methods analysis with weekly panel data. Soc Ment Health. 2025;0(0):21568693251345117.

[49] Bauer AM, Chen C-N, Alegría M. Prevalence of physical symptoms and their association with race/ethnicity and acculturation in the United States. Gen Hosp Psychiatry. 2012;34(4):323–331. doi: 10.1016/j.genhosppsych.2012.02.007. 22460006 PMC3383871

[50] Singer S, Sievers L, Scholz I, et al. Who seeks psychodynamic psychotherapy in community-based practices? Patient characteristics examined in a large sample of applications for reimbursement of psychotherapy in Germany. Psychodyn Pract. 2023;29(2):117–135. doi: 10.1080/14753634.2023.2182702.

[51] Durbin A, Moineddin R, Lin E, et al. Mental health service use by recent immigrants from different world regions and by non-immigrants in Ontario, Canada: a cross-sectional study. BMC Health Serv Res. 2015;15(1):336. doi: 10.1186/s12913-015-0995-9. 26290068 PMC4546085

[52] Nohr L, Dumke L, Klein EM, et al. Current outpatient psychotherapeutic care for people with migration and refugee experience in Germany—an overview. Psychother Psychosom Med Psychol. 2024;74:205–213. 38865996 10.1055/a-2304-8902

[53] Bohman H, Låftman SB, Cleland N, et al. Somatic symptoms in adolescence as a predictor of severe mental illness in adulthood: a long-term community-based follow-up study. Child Adolesc Psychiatry Ment Health. 2018;12(1):42. doi: 10.1186/s13034-018-0245-0. 30123319 PMC6090675

[54] Creed F. The predictors of somatic symptoms in a population sample: the lifelines cohort study. Biopsychosoc Sci Med. 2022;84:1056–1066. 10.1097/PSY.0000000000001101 35797562

[55] Leathers C, Kroenke K, Flanagan M, et al. Somatic, anxiety, and depressive (SAD) symptoms in young adult Latinx immigrants: prevalence and predictors. J Immigr Minor Health. 2021;23(5):956–964. doi: 10.1007/s10903-021-01218-3. 34043112

[56] Alegría M, Álvarez K, DiMarzio K. Immigration and mental health. Curr Epidemiol Rep. 2017;4(2):145–155. doi: 10.1007/s40471-017-0111-2. 29805955 PMC5966037

[57] Raemen L, Claes L, Verschueren M, et al. Personal identity, somatic symptoms, and symptom-related thoughts, feelings, and behaviors: exploring associations and mechanisms in adolescents and emerging adults. Self Identity. 2023;22(2):155–180. doi: 10.1080/15298868.2022.2063371.

[58] Dreher A, Hahn E, Diefenbacher A, et al. Cultural differences in symptom representation for depression and somatization measured by the PHQ between Vietnamese and German psychiatric outpatients. J Psychosom Res. 2017;102:71–77. doi: 10.1016/j.jpsychores.2017.09.010. 28992900

[59] Dere J, Sun J, Zhao Y, et al. Beyond “somatization” and “psychologization”: symptom-level variation in depressed Han Chinese and Euro-Canadian outpatients. Front Psychol. 2013;4:377. doi: 10.3389/fpsyg.2013.00377. 23818884 PMC3694214

[60] Zimmer Z, Fraser K, Grol-Prokopczyk H, et al. A global study of pain prevalence across 52 countries: examining the role of country-level contextual factors. Pain. 2022;163(9):1740–1750. doi: 10.1097/j.pain.0000000000002557. 35027516 PMC9198107

[61] Schenk L, Neuhauser H. Methodische Standards für eine migrantensensible Forschung in der Epidemiologie. Bundesgesundheitsblatt Gesundheitsforsch Gesundheitsschutz. 2005;48(3):279–286. doi: 10.1007/s00103-004-0995-0. 15768300

[62] Craig KD, Holmes C, Hudspith M, et al. Pain in persons who are marginalized by social conditions. Pain. 2020;161(2):261–265. doi: 10.1097/j.pain.0000000000001719. 31651578 PMC6970566

[63] Löwe B, Levenson J, Depping M, et al. Somatic symptom disorder: a scoping review on the empirical evidence of a new diagnosis. Psychol Med. 2022;52(4):632–648. doi: 10.1017/S0033291721004177. 34776017 PMC8961337

